# Nanomechanical Characterization of Ovarian Cancer Cell Lines as a Marker of Response to 2c Treatment

**DOI:** 10.3390/ijms24087230

**Published:** 2023-04-13

**Authors:** Domenico Tierno, Eros Azzalini, Rossella Farra, Sara Drioli, Fulvia Felluga, Marco Lazzarino, Gabriele Grassi, Barbara Dapas, Serena Bonin

**Affiliations:** 1 Department of Medical Sciences (DSM), University of Trieste, 34149 Trieste, Italy; tiernodomenico@gmail.com (D.T.); eazzalini@units.it (E.A.); 2 Department of Life Sciences (DSV), University of Trieste, 34128 Trieste, Italy; farrarossella@libero.it (R.F.); ggrassi@units.it (G.G.); bdapas@units.it (B.D.); 3 Department of Chemical and Pharmaceutical Sciences (DSCF), University of Trieste, 34127 Trieste, Italy; sdrioli@units.it (S.D.); ffelluga@units.it (F.F.); 4 Consiglio Nazionale delle Ricerche, Istituto Officina dei Materiali (IOM), 34149 Trieste, Italy; lazzarino@iom.cnr.it

**Keywords:** stiffness, AFM, ovarian cancer cell lines, heterogeneity, resistance, invasiveness, 2c

## Abstract

Epithelial ovarian cancers (EOCs) are a heterogeneous group of tumors with different molecular and clinical features. In past decades, few improvements have been achieved in terms of EOC management and treatment efficacy, such that the 5-year survival rate of patients remained almost unchanged. A better characterization of EOCs’ heterogeneity is needed to identify cancer vulnerabilities, stratify patients and adopt proper therapies. The mechanical features of malignant cells are emerging as new biomarkers of cancer invasiveness and drug resistance that can further improve our knowledge of EOC biology and allow the identification of new molecular targets. In this study, we determined the inter and intra-mechanical heterogeneity of eight ovarian cancer cell lines and their association with tumor invasiveness and resistance to an anti-tumoral drug with cytoskeleton depolymerization activity (2c).

## 1. Introduction

Ovarian cancer (OC) is the seventh most common cancer and the fifth cause of cancer death among females [1]. In 2018, as reported by the Global Cancer Observatory [2], OCs accounted for 3.4% of all female cancer diagnoses and 4.4% of all female cancer deaths. Epithelial ovarian cancers (EOCs) are the most common and deadly OCs as a heterogeneous group of neoplasms with different behavior, epidemiology, molecular profile and prognosis [3]. Among EOCs, high-grade serous carcinoma (HGSOC, 70% of all EOC cases), low-grade serous carcinoma (LGSOC, <5% of all EOCs), endometrioid carcinoma (10%), clear cell carcinoma (10%) and mucinous carcinoma (3%) are by far the most common and representative subtypes [4].

Despite the efforts in understanding EOC biology, the carcinogenesis of these tumors is poorly understood. Moreover, few improvements in patients’ survival have been achieved over the past 40 years due to late diagnosis, lack of effective treatments and tumor heterogeneity [5]. A deeper understanding of the biological and physical diversity of tumors and their relationships to the response to different therapies are therefore needed and of increasing importance. This is all the more necessary for in vitro studies where cultured cells still represent one of the main sources of knowledge in EOC biology.

In recent years, cell biomechanics have been used for a more comprehensive characterization of EOCs’ heterogeneity both at the cellular and tissue level [6,7]. In 2012, by linking nanomechanical signatures to histological characteristics, Plodinec and colleagues showed that invasive breast cancer tissues were softer than the corresponding benign and healthy counterparts [8]. Other studies, instead, have highlighted how peculiar populations with different mechanical proprieties may be present within the same cell line or tumor tissue. In particular, the distribution of stiffness values may present a bimodal pattern indicative of two cancer subpopulations, with a lower peak associated with cell invasiveness and a higher one associated with drug resistance [9]. Accordingly, cellular stiffness (Young’s modulus) can be used as a new and efficient EOC biomarker to better characterize tumor heterogeneity and response to treatments.

In this regard, non-selective inhibitors of the deubiquitinating enzymes (iDUBs) are an interesting therapeutic option able to act on different cellular pathways. In particular, the DUB inhibitor 2c (also called DU-UC15), developed in the laboratory of bio-organic chemistry from the University of Trieste [10], can trigger a non-specific necrotic pathway involving actin cytoskeleton reorganization [11] that can be investigated by AFM.

In this study, we analyzed the mechanical properties of eight OC cell lines by atomic force microscopy (AFM) as characterization biomarkers of EOC inter and intra-mechanical heterogeneity. The same cell lines were submitted to an invasion assay and treated with 2c to test the association between cellular stiffness, metastatic potential and drug responsiveness. Lastly, the results of 2c treatment were validated by fluorescence staining for F-actin and β-tubulin in order to investigate the effects of 2c in the cytoskeletal organization.

## 2. Results

### 2.1. Ovarian Cancer Cell Lines Display Inter and Intra-Tumoral Heterogeneity at the Biomechanical Level

Cell lines representative of the most frequent epithelial ovarian cancers have been selected and used in the present study. In detail, according to Barnes et al. [12], SKOV3, IGROV1 and OAW42 were classified as non-serous ovarian cancer cell lines (NSOC); TYKNU, TYKNU CpR, HEY and OVCAR8 as low grade serous ovarian cancer (LGSOC) cell lines; while OVCAR4 was indicated as a good model of high grade serous ovarian cancer (HGSOC). The most characterizing results of the study are summarized in Table 1. The AFM analysis of the eight ovarian cancer cell lines showed a widespread distribution of the mean Young’s modulus (from 0.28 ± 0.12 kPa for TYKNU to 1.13 ± 0.50 kPa for OVCAR4) with a statistically significant overall difference, as established by the Kruskal–Wallis test (*p* = 0.0003) (Table 1).

**Figure 1:**
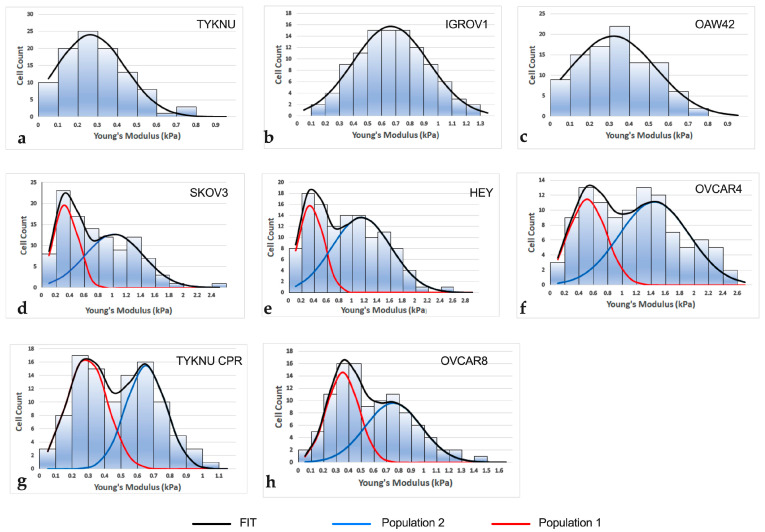
Distribution pattern of single-cell elastic moduli for the analyzed cell lines. Histograms of cell lines with Gaussian stiffness distribution (**a**–**c**). Histograms of cell lines with bimodal stiffness distribution (**d**–**h**). FIT: fitting by peak deconvolution method. Population 1: lowest-stiffness population; Population 2: highest-stiffness population.

To characterize intra-tumor heterogeneity, in each cell line, the distribution of single-cell elastic moduli was analyzed, and the presence of different subpopulations was inferred by applying a peak deconvolution method [13,14]. The analysis returned a unimodal (Gaussian) distribution for OAW42, IGROV1 and TYKNU cells, while OVCAR4, OVCAR8, TYKNU CpR, HEY and SKOV3 had a bimodal curve characterized by two peaks, suggesting the presence of two subpopulations with distinct mechanical properties (Figure 1). On average, the Young modulus of the stiffer population was three times higher than the softer one. The specific stiffness values for each peak are reported in Supplementary Table S1.

Notably, the average elastic modulus of TYKNU cell line was about two times lower than the corresponding cells resistant to cisplatin, namely TYKNU CpR (*p* < 0.0001). Moreover, TYKNU showed a Gaussian profile, while TYKNU CpR had a bimodal pattern where the mean Young’s modulus of the lowest-stiffness population (0.24 ± 0.12 kPa) was very close to that of TYKNU (0.28 ± 0.12 kPa).

### 2.2. Cell Lines Characterization and Invasion Assay

Each cell line was classified according to the morphological criteria reported in Supplementary Table S2. The results showed that the biomechanical features, namely the mean elastic modulus and the distribution pattern, were not associated with the cells’ morphological classification.

An invasion assay was carried out for each cell line to associate their mechanical parameters with the metastatic potential. Table 1 shows the invasiveness score, namely the mean number of invasive cells per optical field.

**Figure 2:**
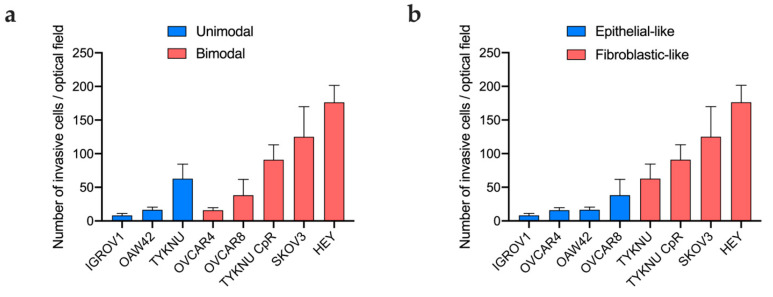
Number of invasive cells for each EOC cell line according to stiffness distribution profile (**a**) and morphological classification (**b**).

Overall, the linear regression analysis of invasion data against the mean Young’s modulus did not return any recognizable pattern (R^2^: 0.05, *p*: 0.6) (Supplementary Figure S1a). However, if only the population with the lowest stiffness in cell lines with bimodal distribution was considered, a significant correlation between the mean stiffness and the invasiveness score was detected (R^2^: 0.49, *p*: 0.05) (Supplementary Figure S1b). Overall, the average invasiveness score was higher in cell lines with bimodal distribution compared to those having a Gaussian distribution (invasiveness score = 89 vs. 29), but the difference was not significant (*p* = 0.2) (Figure 2a).

Morphologically, cell lines with fibroblastic-like morphology had a remarkably high number of invasive cells compared to those with epithelial morphology (*p* = 0.0002; Figure 2b).

### 2.3. Association between Gene Expression and Mechanical Properties and Invasiveness

**Figure 3:**
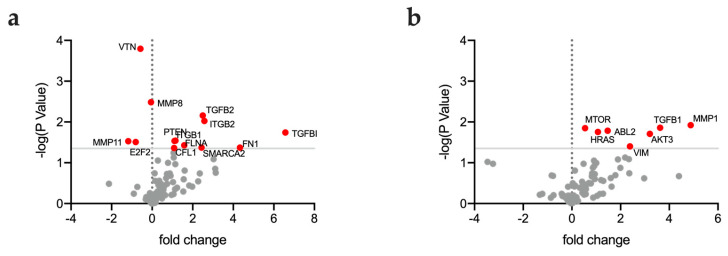
Volcano plots representing differential gene expression in cell lines with unimodal vs. bimodal stiffness distribution (**a**) and low vs. high invasiveness score (**b**). Red dots indicate genes with a significant different expression between groups.

To analyze the relationship between gene expression, the mechanical heterogeneity and invasiveness of the EOC cell lines’ RNAseq data (available for six out eight cell lines) were extracted from CCLE database and submitted to multiple *t*-test analysis. Our results highlighted a different expression pattern between unimodal and bimodal cell lines as shown in Figure 3a. Notably, bimodal cell lines resulted in having a higher expression level of genes involved in cytoskeleton organization and epithelial–mesenchymal transition. As expected, more invasive cell lines characterized by fibroblastic-like morphology expressed higher levels of genes involved in the mTOR pathway and in epithelial–mesenchymal transition, as shown in Figure 3b.

**Figure 4:**
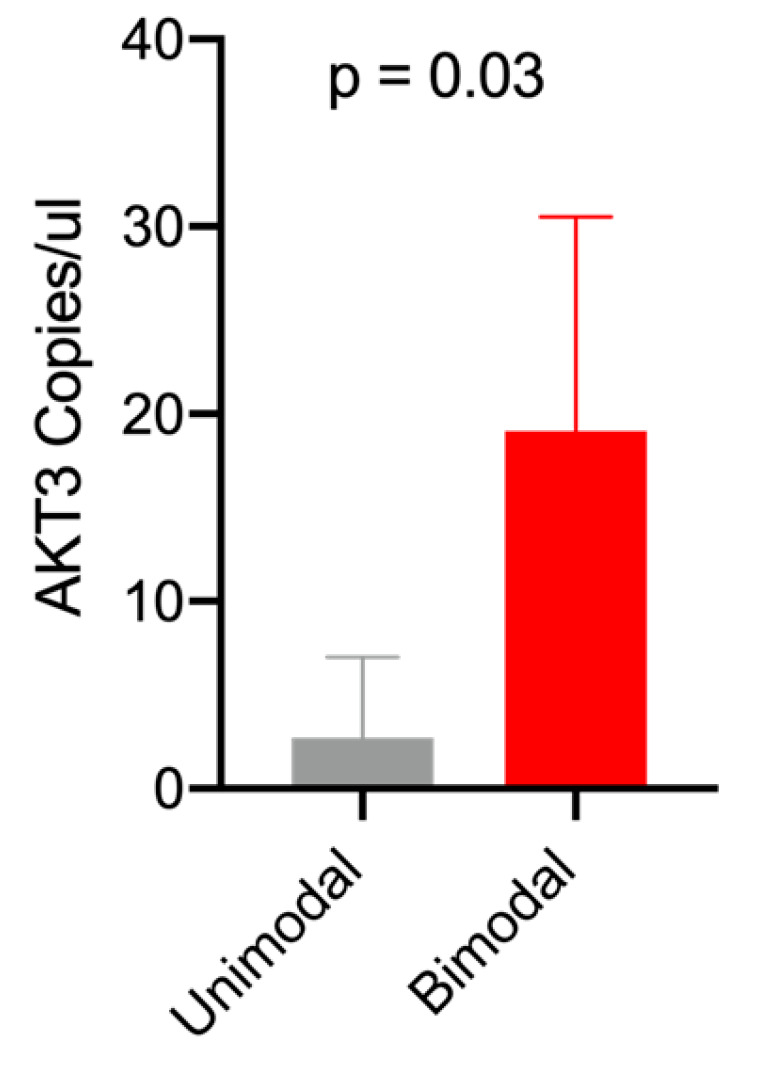
Differential expression of AKT3 mRNA in unimodal and bimodal EOC cell lines.

Gene expression data obtained by ddPCR in all the cell lines investigated in the present study returned a higher expression level of AKT3 transcripts in bimodal cell lines, which were, in general, characterized by higher stiffness values (Figure 4).

### 2.4. Mechanical Heterogeneity of EOC Cell Lines Is Associated with 2c Sensitivity

To evaluate the stiffness as a possible biomarker of response to 2c treatment, the IC50 value was measured in each ovarian cancer cell line and then related to the corresponding elastic modulus.

**Figure 5:**
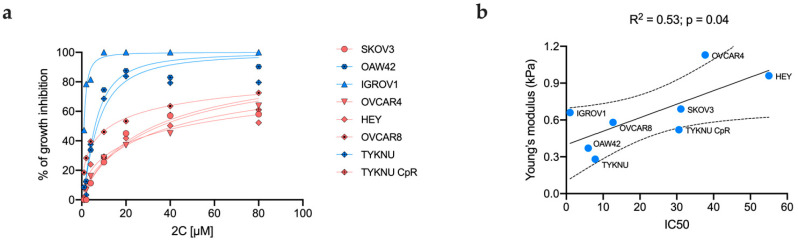
MTT assay and correspondent IC50 values according to cell line’s stiffness distribution pattern (blue = unimodal; red = bimodal) (**a**). Scatterplot of the average elastic modulus in function of the IC50 value (**b**).

EOC cell lines exhibited a broad spectrum of sensitivity, with IC50 values ranging from 1.01 μM for IGROV1 to 55 μM for HEY (Table 1). The 2c response according to the stiffness distribution profile showed that cell lines with bimodal distribution were remarkably more resistant to 2c than those characterized by a unimodal pattern (*p* < 0.0001) (Figure 5a). Accordingly, the TYKNU CpR cells, characterized by a bimodal curve, were more resistant to 2c when compared to TYKNU, which were more sensitive and had a Gaussian profile (IC50 = 30.58 μM vs. 7.86 μM, respectively).

The regression analysis returned a significant correlation between the IC50 value and the average Young modulus (R^2^: 0.53, *p*: 0.04) (Figure 5b). Notably, the goodness of fit slightly improved when considering only the highest-stiffness population in cell lines with a bimodal distribution (R^2^: 0.55, *p*: 0.03), while the correlation was no more significant when considering only the lowest one (R^2^: 0.3, *p*: 0.2), suggesting a possible implication of the stiffer population in the resistance to 2c (Supplementary Figure S2a,b).

No evident association, instead, was observed between the sensitivity to 2c and the morphological classification.

### 2.5. The 2c Treatment Modulates the Mechanical Behavior of Ovarian Cancer Cell Lines

Cells lines were split into two aliquots and treated with 20 µM of VV1 inactive drug control or 2c and after 7 h submitted to AFM. The mean Young moduli, before and after treatment, were recorded, and the coefficient of variation (CV%) of cell stiffness was calculated to investigate the drug effects on the mechanical proprieties of the cells. In addition, the IC50 and CV% parameters were dichotomized in “high” and “low” groups according to their median value (IC50 = 22 and CV% = 42) and used for further analyses.

**Figure 6:**
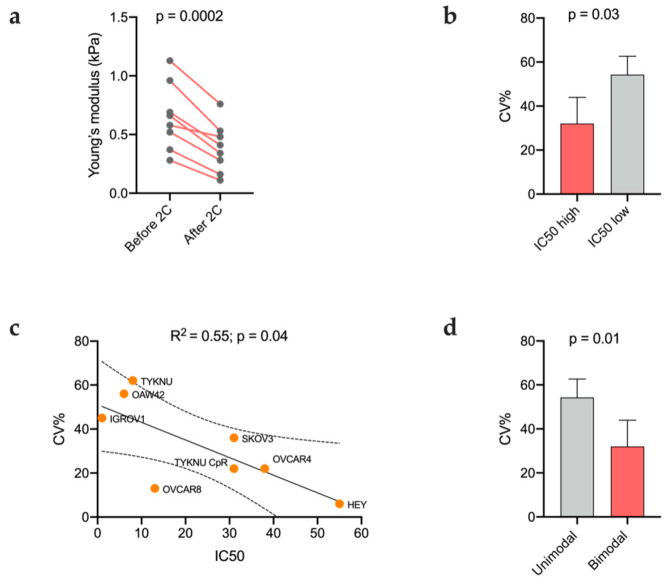
Modulation of cellular stiffness in response to 2c. Changes in the mean cellular stiffness after 2c administration in the eight EOC cell lines (**a**); coefficient of variation (CV%) of cellular stiffness in cell lines with high and low IC50 values (**b**); scatterplot of the IC50 value in function of the coefficient of variation (CV%) of the elastic moduli of the stiffer population in cell line with bimodal pattern (**c**); coefficient of variation (CV%) of cellular stiffness in cell lines with bimodal and unimodal stiffness distribution (**d**).

The treatment with 2c led to an overall decrease in the average elastic modulus in all cell lines when compared to the untreated ones with a statistically significant difference between the two groups (*p* = 0.0002; Figure 6a and Appendix A Table A1). As expected, the CV% was remarkably lower in cell lines with higher resistance to 2c compared to the sensitive ones (*p* = 0.03; Figure 6b), but the average IC50 and CV% values were not correlated. Nevertheless, when considering only the CV% of the stiffer population in cell lines with a bimodal pattern, a significant and linear correlation was obtained (R^2^: 0.55, *p*: 0.04) (Figure 6c). Accordingly, *t*-test comparison showed that cell lines with a bimodal pattern varied their stiffness to a significantly lesser extent than those having a Gaussian distribution (mean CV = 32% vs. 54%, respectively; *p* = 0.01) (Table 1 and Figure 6d).

**Figure 7:**
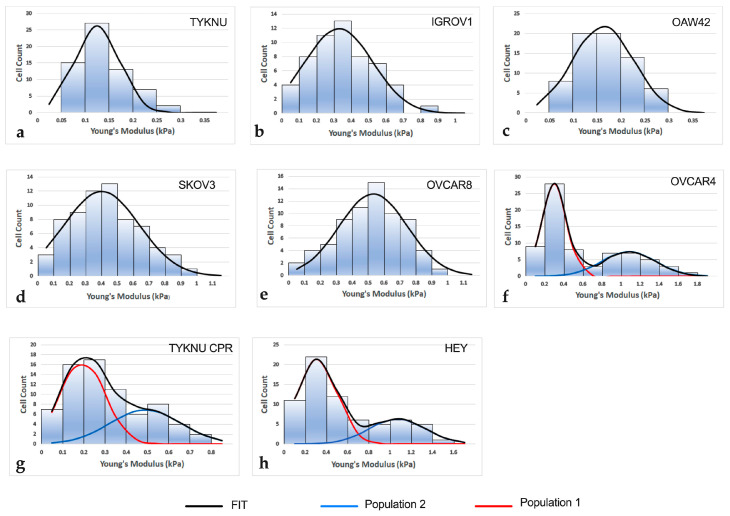
Histograms of cell lines with Gaussian stiffness distribution after 2c treatment (**a**–**e**). Histograms of cell lines with bimodal stiffness distribution after 2c treatment (**f**–**h**). FIT: fitting by peak deconvolution method. Population 1: lowest-stiffness population; Population 2: highest-stiffness population.

Concerning the effect of 2c on the distribution of single-cell elastic moduli, all cell lines with unimodal patterns retained a Gaussian profile after 2c treatment, while those with a bimodal pattern displayed a more heterogeneous behavior. In particular, HEY, OVCAR4 and TYKNU CpR continued to be non-normal after 2c administration, while SKOV3 and OVCAR8 lost their bimodality assuming a Gaussian curve. (Figure 7). Overall, the effect of 2c treatment on the three cell lines retaining a bimodal pattern was more evident in the lowest-stiffness population compared to the highest one (mean CV = 32% vs. 16%, respectively), suggesting a possible role for the latter in the resistance to therapy (Supplementary Table S3).

### 2.6. The 2c Is Quickly Incorporated into Cells

To evaluate the 2c internalization and distribution inside the cell, OVCAR4 and HEY cells were treated with 2c-F2 (fluorescent label) and observed after 7 h. Those cell lines were chosen as representative of cells with the highest IC50 (HEY) and intermediate (OVCAR4). TYKNU cell line was excluded from this experiment because of the low IC50 value. The analysis of the fluorescence images showed that 2c-F2 was consistently incorporated at 6 h from administration (Supplementary Figure S3). For both cell lines, the fluorescence intensity inside the cells resulted on average at least 2-fold higher than the one measured in background after 6 h from the treatment (2.76 for OVCAR4 and 3.63 for HEY).

### 2.7. A homogeneous Distribution of F-Actin Cytoskeleton over the Cell Is Associated with Higher Resistance to 2c

Based on the sensitivity to 2c, three cell lines, HEY, TYKNU and OVCAR4, were selected and submitted to immunofluorescence staining for F-actin to analyze stress fibers before and after drug administration. Accordingly, HEY cells had the highest IC50, TYKNU the lowest and OVCAR4 an intermediate value.

Before treatment, the fluorescence intensity of F-actin was more concentrated in the cell center for the TYKNU cell line, the one presenting a unimodal distribution, low stiffness and IC50, while it was more diffuse throughout the cell in OVCAR4 and HEY, the cell lines characterized by a bimodal pattern, high stiffness and IC50. Notably, fiber organization was consistent with the cell line morphological classification: parallel bundles for the TYKNU and HEY (fibroblastic-like morphology) and tangled network for OVCAR4 (epithelial-like morphology) [15].

**Figure 8:**
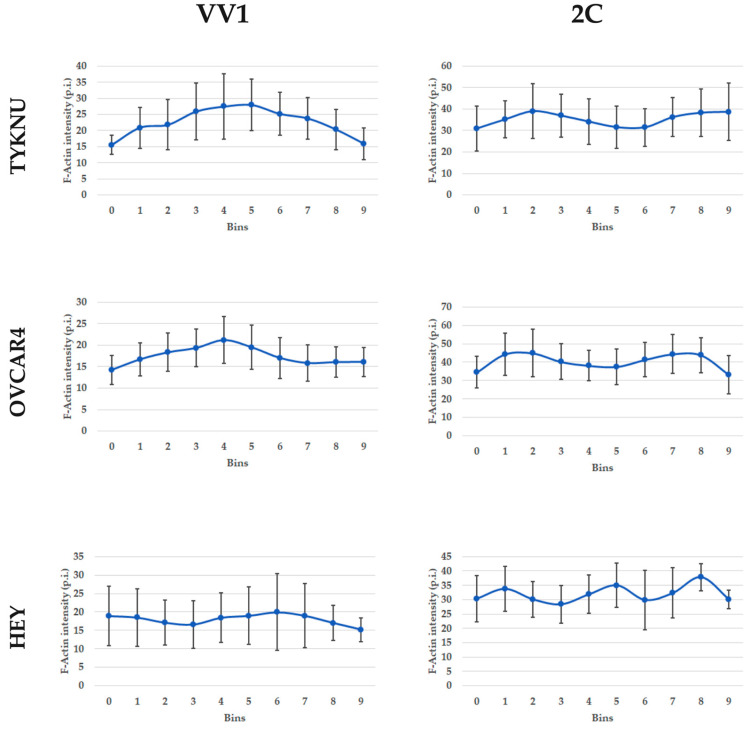
F-actin fluorescence intensity profile for cell lines treated with the inactive control drug VV1 or 2c. X-axis: cell centroid crossline divided into 10 equal sections (bins). The three most external bins (1–3 and 8–10) indicate the cell periphery while the inner ones (4–7) indicate the cell center. Y-axis: average F-actin fluorescence intensity for each bin expressed as mean pixel intensity. Points bars indicate the 95% confidence interval (CI).

**Figure A1:**
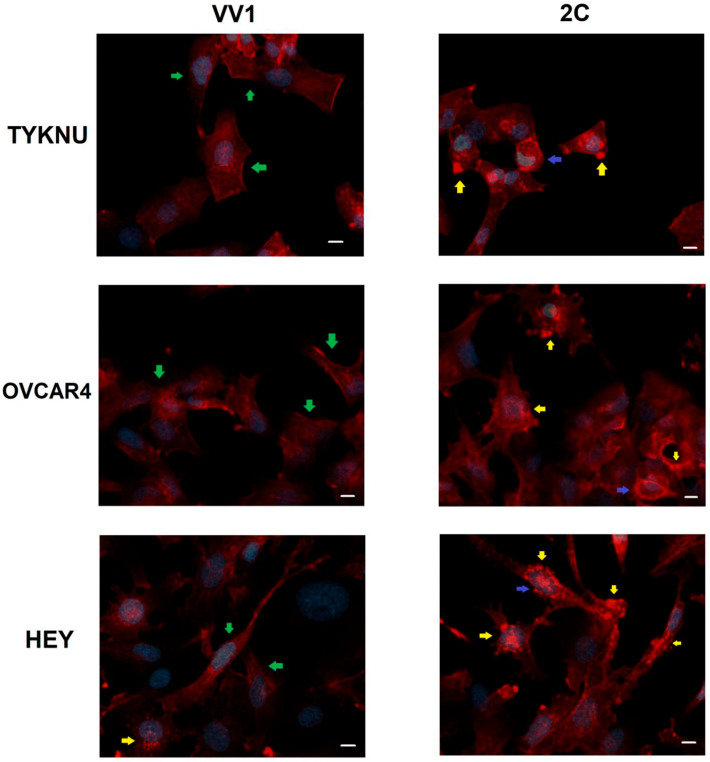
Images of F-actin immunofluorescence staining by phalloidin for TYKNU, OVCAR4 and HEY cell lines treated with VV1 (negative control) and 2c. In blue, the DAPI-marked nuclei. Green arrows: stress actin fibers; Yellow arrows: high-intensity spots; Blue arrows: high-intensity rings. Scale bar = 10 µm.

After 2c treatment, all three cell lines displayed disrupted actin cytoskeletons, with the accumulation of high-intensity spots and rings in the cell periphery. This effect was particularly evident for TYKNU and OVCAR4, while in HEY the intensity profile was more heterogeneous (Appendix A Figure A1). The specific variations in fluorescence intensity measured from the cell center to its periphery are depicted in Figure 8.

### 2.8. The Microtubules Organization Is Disrupted by 2c

To further investigate the effects of 2c on cytoskeletal organization, immunofluorescence staining for β-tubulin was carried out in TYKNU, OVCAR4 and HEY before and after 2c treatment.

Cell lines treated with the inactive form of 2c (VV1) showed a “bush” organization characterized by microtubule filaments from the cell center to the periphery. OVCAR4 displayed higher intensity in the cell center compared to the border, while HEY and TYKNU presented a more diffuse pattern all over the cell.

**Figure 9:**
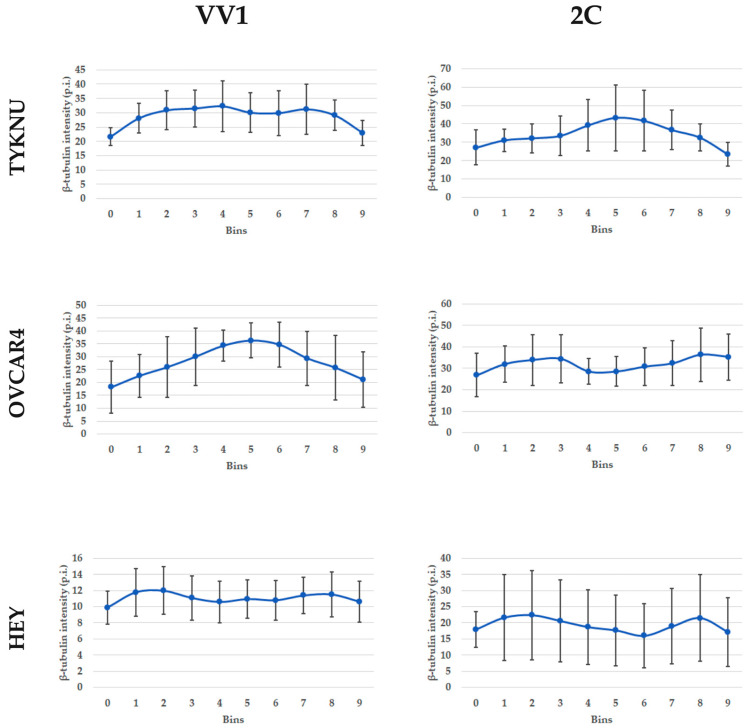
β-tubulin fluorescence intensity profile of cell lines treated with VV1 and 2c. X-axis: cell centroid crossline divided into 10 equal sections (bins). The three most external bins (1–3 and 8–10) indicate the cell periphery while the inner ones (4–7) indicate the cell center. Y-axis: average β-tubulin fluorescence intensity for each bin expressed as mean pixel intensity. Points bars indicate the 95% confidence interval (CI).

**Figure A2:**
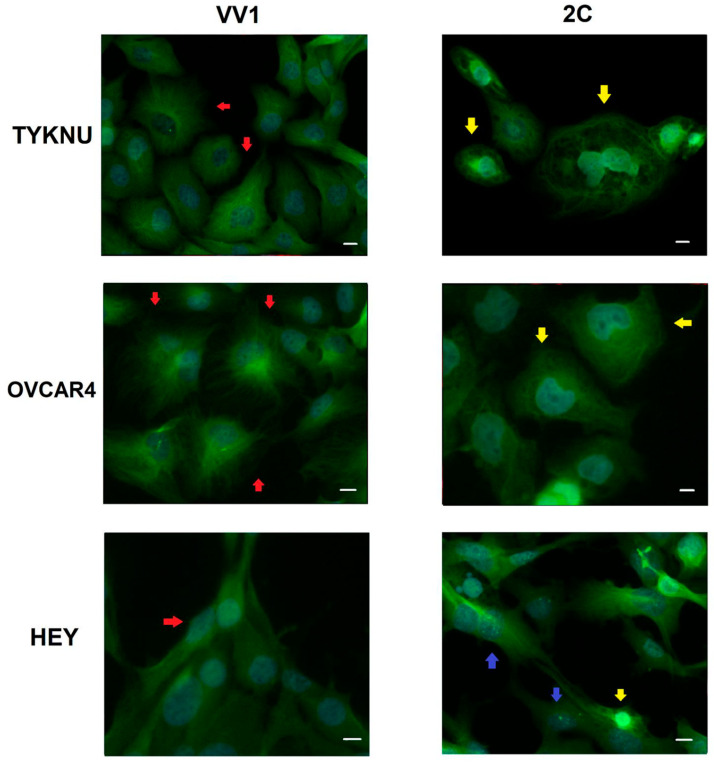
Images of β-tubulin immunofluorescence staining for TYKNU, OVCAR4 and HEY cell lines treated with VV1 (negative control) and 2c. In blue, the DAPI-marked nuclei. Red arrow: ”bush” microtubules network; Yellow arrow: disrupted microtubules; Blue arrow: Nuclear high-intensity point. Scale bar = 10 µm.

On the contrary, after treatment with 2c, cells presented a sharp splitting of the microtubule network, which appeared disorganized in filaments throughout the cell. In particular, the fluorescence intensity of TYKNU cells was higher in the cell center compared to the border, while in OVCAR4 and HEY it was prominent on the cell periphery (Appendix A Figure A2). The spatial variations of β-tubulin intensity are depicted in Figure 9.

## 3. Discussion

In this study, AFM was applied to investigate the inter and intra-mechanical heterogeneity of eight EOC cell lines and their associations with tumor invasiveness and response to 2c treatment. Tumor heterogeneity was inferred by applying a fitting deconvolution method on single-cell stiffness distribution. The resulting curves showed mainly two different patterns: a unimodal distribution in three cell lines and a bimodal pattern in five. The latter was characterized by two main peaks indicative of two populations with different mechanical features.

Intra-mechanical heterogeneity can be an important marker of drug sensitivity.

Our results on the IC50 analysis for 2c have indeed shown that cell lines with a bimodal pattern are more resistant to treatment than those with a unimodal pattern. This was particularly evident for TYKNU and its cisplatin-resistant subline, TYKNU CpR, which were, respectively, unimodal and bimodal. In addition, TYKNU CpR was significantly stiffer compared to TYKNU. This is in agreement with Raudenska et al., who reported an enhancement in cellular stiffness mediated by cisplatin treatment in prostate cancer cell lines [16]. Notably, the stiffness value of TYKNU CpR softer subpopulation was comparable with that of TYKNU. This could indicate that the presence of the highest stiffness population may be due to a clonal selection induced by the treatment with cis-platinum of the sensible cell line (TYKNU), which was treated with step-wise increasing concentration of drug for obtaining the resistant one (TYKNU CpR). In agreement with this, we found a significant linear correlation between IC50 and the average Young modulus when considering the stiffer subpopulation and not the softer one in cell lines with a bimodal pattern.

Intra-mechanical heterogeneity of cell lines has already been correlated with drug sensitivity in triple-negative breast cancer cell lines by optical stretching [17]. By a machine learning approach, two different stiffness clusters within the same cancer cell line have been identified and possibly linked to metastatic potential [17].

In ovarian cancer lines SKOV3 and OVCAR5, Sharma and co-authors reported a bimodal distribution of the Young modulus by AFM in the cis-platinum resistant replicates, namely OVCAR5-CisR and SKOV3-CisR. In agreement with us, the stiffness of the softer subpopulation in the resistant subline was similar to the corresponding sensitive ones [9].

Despite that several studies have indicated that low stiffness was associated with higher tumor invasiveness [8,18,19], our analysis of invasion data in function of the mean Young’s modulus did not show a strong correlation. Of note, cell lines with bimodal stiffness distribution pattern were characterized by two subpopulations with different mechanical properties, and possibly, according to the literature, we can hypothesize that the softer subpopulation could be more invasive than the stiffer one [20,21]. Accordingly, when we considered only the average Young’s moduli of the lowest-stiffness population in cell lines with a bimodal distribution, a strong and significant correlation between the mean stiffness and the average number of invasive cells was recorded. In particular, the decrement in stiffness was correlated to a linear increment in invasiveness supporting that only the softer population could have the metastatic potential to invade. Moreover, it is important to consider that OVCAR4 and OVCAR8 cell lines, which had a bimodal distribution of Young moduli, had a relatively low invasion capacity, suggesting that the metastatic potential was not only related to the stiffness distribution pattern but also to the absolute value of Young’s modulus.

In our study, clinical data of the patients from which the eight EOC cell lines were isolated further support the potential of intra-mechanical heterogeneity as a marker of drug responsiveness. Cell lines with a bimodal pattern were indeed derived from patients with platinum or Adriamycin resistance, while three out of four cell lines with unimodal profiles were obtained from patients with no reported drug resistance [22,23,24,25,26,27,28].

The 2c is a non-selective and irreversible inhibitor of isopeptidases of possible use in tumor treatment [11]. Its principal targets are the DUBs, a class of isopeptidases involved in the proteasome-mediated protein degradation pathway. The inhibition of DUBs leads to a proteo-toxic shock of tumor cells, which are extremely dependent on the functional protein degradation pathway because of the high number of aberrated proteins. Some efforts have indicated a parallel activity of this drug in triggering a cellular necrotic pathway that involves cytoskeletal reorganization. This is due to the wide range of proteins that can be targeted by the inhibitory activity of 2c [11,29]. As ubiquitin-specific peptidase 5 has been shown to promote ovarian cancer cell proliferation, 2c can represent a possible new treatment for EOC [30]. The possible utility of 2c in ovarian cancer can also be related to its activity on AKT molecules [29], which have already been related to patients’ survival, tumor morphology and BRCA1 expression [31]. Accordingly, our data obtained by droplet digital PCR analysis returned higher expression levels of the AKT3 gene in bimodal cell lines compared to the unimodal ones, further stressing the role of AKT isoforms in mediating the resistance to therapy.

Our results showed that 2c administration led generally to a significant decrease in cell stiffness, which well fits with its action on the depolymerization of the F-actin network by activation of Cofilin-1 [11]. Those results are also supported by the immunofluorescence of F-actin, which showed a variation of the staining after 2c treatment. F-actin signal decentralized to the periphery of the cell after 2c treatment, in agreement with other drugs destabilizing the cytoskeleton [32]. Similar to F-actin fibers, the 2c treatment also varied the microtubules network as evidenced by β-tubulin stain, which spreads in disorganized filaments over the cell consistently to the mechanisms of other microtubule destabilizing drugs [33]. Therefore, this result highlights that the 2c also alters other components of the cytoskeleton. Nevertheless, the variation in β-tubulin staining and consequently in microtubules seems to be associated with the morphological classification of the cell lines, namely epithelial vs. fibroblastic, rather than the cell stiffness or the IC50. Therefore, the disruption or chemical destabilization of microtubules did not seem to directly affect cell elasticity as already shown [34].

Fibroblastic-like cell lines were characterized by a remarkable higher number of invasive cells than those with epithelial morphology. It is well known that cancer invasion ability depends also on the degree of epithelial differentiation within the tumor: poorly differentiated tumors are usually more invasive than well-differentiated ones. For this reason, cancer cells need to undergo an epithelial–mesenchymal transition (EMT) to lose adhesion and epithelial morphology and to assume, in this way, mobility with a mesenchymal–fibroblast morphology [35]. Accordingly, in agreement with other authors, our results showed that genes involved in EMT were significantly more expressed in cell lines characterized by higher invasiveness score and fibroblastic-like morphology compared to less invasive ones and those with epithelial-like morphology [36,37,38,39,40].

Taken this evidence, our results on invasion assay in function of morphological classification are in agreement with the fundamental processes underlining the metastatic diffusion.

Cell lines with unimodal and bimodal stiffness distribution seem to modulate differently their elastic proprieties after 2c administration. Our results indicated a tight association between the stiffness distribution pattern (bimodal/unimodal), the CV% of cellular stiffness after 2c treatment and the IC50 value. The CV% was remarkably lower in cell lines with higher resistance to 2c compared to the sensitive ones. Therefore, the softer the cells are after treatment the higher the response. In agreement with this, we found that cell lines with bimodal pattern, which displayed a lower CV% compared to the unimodal ones, were also more resistant to 2c. Results from RNAseq analysis highlighted a significantly higher expression of genes involved in cellular stiffening, 2c response and EMT in EOC cell lines with bimodal pattern as reported in ovarian cancers as well as in other cancer types during progression [41,42,43,44,45]. We can also speculate that the higher resistance found in cell lines with bimodal pattern can be mainly attributable to the highest stiffness population rather than the softest one since the CV% after treatment was significantly lower in the first compared to the latter. Accordingly, we found that the overall correlation between IC50 and CV% was statistically significant only when considering the stiffer subpopulation in the bimodal group. Moreover, cell lines with unimodal distribution retained their pattern even after treatment, while SKOV3 and OVCAR8, which had a bimodal distribution, after treatment moved to unimodal stiffness distribution, highlighting possibly that 2c is an effective drug against one cell population. Although 2c is a non-selective isopeptidase inhibitor acting on different targets, our results show that at least in EOC the CV% of the Young modulus can represent a possible surrogate biomarker for 2c therapeutic response.

Overall, this study highlights that intra-mechanical heterogeneity can be a possible marker for 2c response and cell invasiveness. We acknowledge however that our results are based solely on cell line models; therefore, they should be validated in a broader number of cell lines/primary cell culture as well as with in vivo experiments allowing a deepening of tumor features for clinically relevant conclusions.

## 4. Materials and Methods

### 4.1. Ovarian Cancer Cell Lines

Eight epithelial ovarian cancer cell lines were analyzed in the present study. SKOV3 and IGROV1 cell lines were generously provided by Prof. G. Ricci (Institute for Maternal and Child Health–IRCCS “Burlo Garofolo”, Trieste, Italy), while HEY, OVCAR8, OVCAR4, TYKNU, TYKNU CpR and OAW42 cell lines were provided by Prof. O.M. Carpen (University of Helsinki, Helsinki, Finland). The TYKNU CpR is a cis-platinum resistance cell line derived from the treatment of TYKNU cell line with a stepwise concentration of cis-platinum [22].

The HEY, OVCAR4 and OVCAR8 were cultured in RPMI 1640 medium (EuroClone S.p.A., Milan, Italy) supplemented with 10% fetal bovine serum (FBS, Sigma-Aldrich Co., St. Louis, MO, USA), 1% Streptomycin-Penicillin 25× (EuroClone S.p.A., Milan, Italy) and 1% Gentamicin (EuroClone S.p.A., Milan, Italy). The SKOV3 and OAW42 were grown in high-glucose DMEM medium (Sigma-Aldrich Co., St. Louis, MO, USA) supplemented with 10% FBS, 1% streptomycin-Penicillin 25× and 1% Gentamicin. Finally, the TYKNU and TYKNU CpR cell lines were cultured in EMEM medium (Sigma-Aldrich Co., St. Louis, MO, USA) supplemented with 10% FBS, 1% streptomycin-Penicillin 25× and 1% Gentamicin, while the IGROV1 cells were grown in DMEM: F12 (1:1) medium supplemented (Sigma-Aldrich Co., St. Louis, MO, USA) with 10% FBS, 1% streptomycin-Penicillin 25× and 1% Gentamicin. All cell lines were incubated at 37 °C in a humidified atmosphere of 5% CO_2_ and were plated in filtered cap flasks (T25 or T75, Corning^®^, Corning, NY, USA). To discriminate between the fibroblastic-like and epithelial-like morphology the “Aspect ratio” and “Circularity” of cells were investigated as already described [46]. Cells with circularity < 0.5 and aspect ratio > 2.5 were considered fibroblastic-like, otherwise, epithelial-like. Accordingly, the SKOV3, HEY, TYKNU and TYKNU CpR were classified as fibroblast-like/spindle morphology cell lines while the IGROV1, OAW42, OVCAR8, and OVCAR4 as cell lines with epithelial-like morphology (Supplementary Table S2). The rounded morphology was excluded since each cell line grew in adherence.

### 4.2. AFM Measurements

For each cell line, AFM measurements were carried out in about 100 cells in three different experiments. For AFM measurements an MF3D-Bio AFM (Asylum Research, Santa Barbara, CA, USA) with an inverted optical microscope (Nikon, Melville, NY, USA) was used. The mechanical measurements were performed using the cantilevers PNP-TR-TL-50 (Nano World, Neuchâtel, Switzerland) with a triangular shape and nominal elastic constant of 0.32–0.08 N/m. The cantilevers were functionalized with silica beads with a diameter of 4.5 μM to allow cell microindentation and decrease simultaneously the risks of sample damage. As the first step, cells were seeded on a glass coverslip and analyzed by AFM within 2–3 days from seeding. Measurements were carried out in complete medium warmed at 37 °C to keep cells healthy during the analysis. For each analyzed cell at least five force-displacement curves at different locations in the cell peri-nuclear region were collected. The microindentation was performed at an indentation velocity of 2 μm/s, slow enough to avoid hydrodynamic effects, and a force-distance of 5 μm/s, large enough to ensure the tip will be fully detached from the cell between indentation instances. Moreover, the deflection trigger point was settled at 2 nN to avoid cell damage [47]. The recorded force–displacement curves were fitted with the Hertz model to obtain the local cell surface elasticity or “stiffness” (Young’s modulus, E). The fitting was carried out at a maximum indentation of 500 nm. This value allows significant indentation of cells with different heights and stretching remaining simultaneously within the linear viscoelasticity.

### 4.3. Invasion Assay

The metastatic potential of the cell lines was assessed using a cell culture insert (8 µm pore size, 24-well format, Cell Invasion Assay Kit Cat. CHEMICON, No. ECM550). For each cell line, a cell suspension aliquot in serum-free medium containing 0.9 × 10^6^ cells/mL was injected into the cell culture insert. After 72 h of incubation, the non-invading cells that had not moved to the underlying well were washed out from the insert, while invading ones were stained and counted through an optical microscope following manufacturer instructions. The average invading cell number was achieved by cell count of at least three optical fields at 10× magnification.

### 4.4. Gene Expression Analysis

To associate mechanical and invasiveness data with gene expression analysis, RNAseq data were extracted from the CCLE dataset http://sites.broadinstitute.org/ccle/ (accessed on 4 April 2023) for 6 out 8 cell lines, namely IGROV1, OAW42, OVCAR8, OVCAR4, SKOV3, TYKNU. In detail, genes involved in cytoskeleton organization, cell cycle control, epithelial mesenchymal transition, invasiveness and response markers to 2c were exported and analyzed. Overall, 80 gene profiles were analyzed (Supplementary Table S4). For the final analysis, data were normalized against the expression level of 3 housekeeping genes, namely HPRT1, HMBS and PPIB.

For a limited number of genes (AKT1, AKT2, AKT3, PI3KCB, PTEN, HER2, CCNE1, RB1, CDK2), the expression analysis was performed by RT-ddPCR using 1 ng of cDNA. RT-ddPCR analyses were carried out as already reported [31], with the difference that RT reaction was carried out using oligo dT priming. PCR conditions as well as primer sequences can be retrieved in previously published reports [31,48]. Data were normalized against the expression level of 3 housekeeping genes, namely HPRT1, HMBS and PPIB.

### 4.5. 2c Treatment

**Figure A3:**
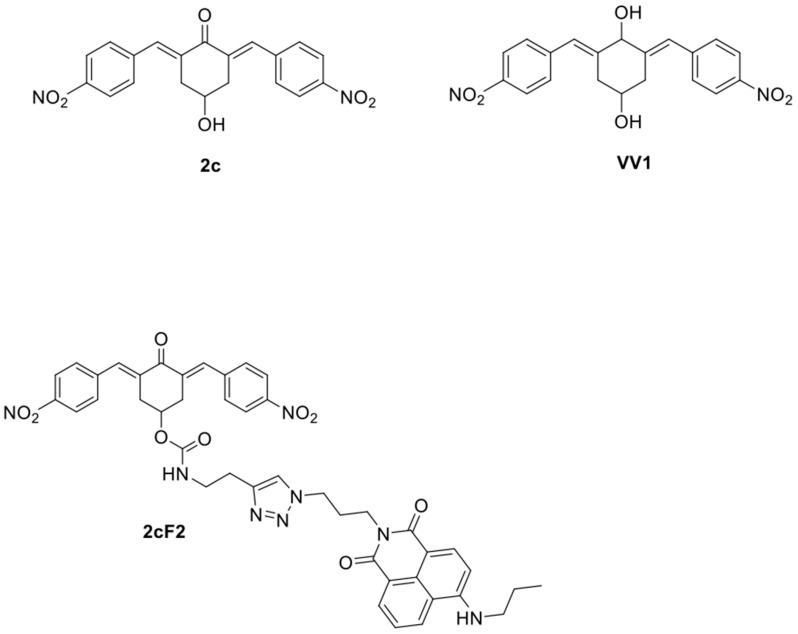
Structure of 2c and its derivatives used in the present study, namely VV1 the inactive form used as negative control and 2cF2, the 2c labeled with the fluorophore 4-propylamino-N-allyl-1,8-naphthalimide used in the uptake analysis.

Each cell line was treated for 24 h with increasing concentrations of 2c: 1–2–4–10–20–40–80 μM (in Figure A3, 2c structure is reported). Afterward, an assessment of cell viability for each concentration was performed by MTT assay to obtain the IC50. Subsequently, cells were treated with 20 μM of 2c and analyzed by AFM after 7 h. To compare results, AFM experimental set-up was the same as the one used for untreated cells. For each cell line, around 70 cells were indented in different spots of their peri-nuclear region.

### 4.6. 2c Uptake Analysis

The 2c uptake assay was performed using the 2c labeled with the fluorophore 4-propylamino-N-allyl-1,8-naphthalimide (F2, λ emission = 524 nm, green, see Figure A3 for its structure). The 2c uptake was investigated after 6 and 24 h of drug administration. OVCAR4 and HEY cells were treated with 2 μM of 2c-F2 and fixed in 4% paraformaldehyde for 15–20 min after the two-time checkpoints. After fixation, the coverslips were treated with DAPI and assembled on a microscope slide. Finally, fluorescence images were obtained (Leica DM2000, Leica Microsystems, Wetzlar, Germany) and the fluorescence intensity of at least 30 cells for each cell line was achieved by ImageJ.

### 4.7. F-Actin and β-Tubulin Stainings

Actin network fluorescence staining was obtained using phalloidin conjugated with tetramethylrhodamine (TRITC, λ emission = 565 nm, ActinRed™ 555, Thermo Fisher Scientific, Waltham, MA, USA). OVCAR4, HEY and TYKNU cells were split into two populations for the treatment with 20 μM 2c and VV1 (see Figure A3 for its structure), the inactive isoform of 2c used as negative control. After 24 h, cells were fixed, permeabilized and treated with 2 drops of phalloidin-TRITC for ml of media. After an incubation of 30 min and washing with PBS 1×, cell lines images were taken at the fluorescence microscope (Leica DM2000) at 20× and 40× magnification and analyzed by ImageJ.

As concerns the microtubule network, an immunofluorescence assay for β-tubulin was carried out. Similarly to actin network analysis after splitting cells into two populations for treatment with 2c and VV1, they were fixed after 24 h in 4% paraformaldehyde and 0.15% picric acid. After treatment with 0.1% Triton X 100 and 1% BSA for membrane permeation, fixed cells were treated with the primary antibody for β-tubulin at a concentration of 10 ng/mL for one hour at room temperature. Subsequently, an anti-rabbit secondary antibody labeled with a green fluorophore (Alexa Fluor^®^ 488, λ emission= 519 nm, Thermo Fisher Scientific, Waltham, MA, USA) was used at a concentration of 2 μg/mL for 45 min to detect the primary antibody/β-tubulin complex. After staining with DAPI, the coverslips were assembled on microscope slides and left at 4 °C overnight. Finally, cell line images were taken at the fluorescence microscope (Leica DM2000) at a magnification of 20× and 40× and analyzed by ImageJ 1.53j.

The distribution of F-Actin and β-tubulin over the cell was achieved using a Fiji Macro of ImageJ to analyze the intensity and the distribution of fluorescence inside cells, as previously described [32].

### 4.8. Statistical Analysis

Data were expressed as mean ± standard deviation. To assess the statistical relevance, the comparisons between groups were performed using parametric or non-parametric test according to variables distribution. Pairwise correlation analyses were carried out using Pearson correlation, while linearity was tested by regression analysis. Data distribution was assessed by the Shapiro–Wilk test. Continuous variables such as IC50, invasiveness score and CV% were dichotomized in “high” and “low” groups according to their median value. The association between categorical variables was assessed by Pearson’s chi-squared test. The fluorescence intensity was reported as the mean pixel intensity (m.p.i.), which is the sum of the color intensity values of all pixels in the selected area divided by the number of pixels. All *p*-values values < 0.05 were considered statistically significant. Statistical analyses were carried out using GraphPad Prism 9 (GraphPad Software, Inc., San Diego, CA, USA), R 4.04 and Origin 2021 (OriginLab Corporation, Northampton, MA, USA).

## Figures and Tables

**Table 1:** Summary of biomechanical, invasiveness and 2c resistance features of the analyzed cell lines.

Cell Line	Morphology ^1^	Average Stiffness (E)	Stiffness Distribution	Invasiveness Score ^2^	IC50 (µM)	CV% ^3^
OAW42	E	0.37 ± 0.14	Gaussian	17 ± 4	5.94	56.03
IGROV1	E	0.66 ± 0.21	Gaussian	8 ± 3	1.01	45.25
SKOV3	F	0.69 ± 0.24	Bimodal	125 ± 45	31.11	36.00
HEY	F	0.96 ± 0.58	Bimodal	176 ± 26	55.00	40.81
TYKNU	F	0.28 ± 0.12	Gaussian	63 ± 22	7.86	61.65
TYKNU CpR	F	0.52 ± 0.23	Bimodal	91 ± 22	30.58	42.43
OVCAR8	E	0.58 ± 0.28	Bimodal	38 ± 24	12.69	13.34
OVCAR4	E	1.13 ± 0.50	Bimodal	16 ± 4	37.7	27.69

^1^ E = epithelial-like morphology; F = fibroblastic-like morphology. ^2^ Mean n° of invasive cells/10× optical field. ^3^ Coefficient of variation (%) of Young modulus after 2c treatment.

## Data Availability

Data supporting reported results are available on request to the corresponding authors.

## Supplementary Materials

The following supporting information can be downloaded at: https://www.mdpi.com/article/10.3390/ijms24087230/s1.

## Appendix A

**Table A1:** Average Young’s modulus of cell lines before (E − 2c) and after (E + 2c) 2c treatment and corresponding coefficient of variation (CV%). F: Fibroblast, E: Epithelial.

Cell Line	Morphology	Stiffness Distribution	E − 2c	E + 2c	CV%
OAW42	E	Gaussian	0.37 ± 0.14	0.16 ± 0.04	56.03
IGROV1	E	Gaussian	0.66 ± 0.21	0.34 ± 0.16	45.25
SKOV3	F	Bimodal	0.69 ± 0.24	0.41 ± 0.17	36.00
HEY	F	Bimodal	0.96 ± 0.58	0.53 ± 0.35	40.81
TYKNU	F	Gaussian	0.28 ± 0.12	0.11 ± 0.02	61.65
TYKNU CpR	F	Bimodal	0.52 ± 0.23	0.28 ± 0.09	42.43
OVCAR8	E	Bimodal	0.58 ± 0.28	0.48 ± 0.18	13.34
OVCAR4	E	Bimodal	1.13 ± 0.50	0.76 ± 0.25	27.69

## References

[1] Siegel R.L., Miller K.D., Jemal A. (2019). Cancer statistics, 2019. CA Cancer J. Clin..

[2] Bray F., Ferlay J., Soerjomataram I., Siegel R.L., Torre L.A., Jemal A. (2018). Global cancer statistics 2018: GLOBOCAN estimates of incidence and mortality worldwide for 36 cancers in 185 countries. CA Cancer J. Clin..

[3] Prat J., D’Angelo E., Espinosa I. (2018). Ovarian carcinomas: At least five different diseases with distinct histological features and molecular genetics. Hum. Pathol..

[4] Reid B.M., Permuth J.B., Sellers T.A. (2017). Epidemiology of ovarian cancer: A review. Cancer Biol. Med..

[5] Lheureux S., Gourley C., Vergote I., Oza A.M. (2019). Epithelial ovarian cancer. Lancet.

[6] Azzalini E., Abdurakhmanova N., Parisse P., Bartoletti M., Canzonieri V., Stanta G., Casalis L., Bonin S. (2021). Cell-stiffness and morphological architectural patterns in clinical samples of high grade serous ovarian cancers. Nanomedicine.

[7] Pei W., Chen J., Wang C., Qiu S., Zeng J., Gao M., Zhou B., Li D., Sacks M.S., Han L. (2019). Regional biomechanical imaging of liver cancer cells. J. Cancer.

[8] Plodinec M., Loparic M., Monnier C.A., Obermann E.C., Zanetti-Dallenbach R., Oertle P., Hyotyla J.T., Aebi U., Bentires-Alj M., Lim R.Y. (2012). The nanomechanical signature of breast cancer. Nat. Nanotechnol..

[9] Sharma S., Santiskulvong C., Bentolila L.A., Rao J., Dorigo O., Gimzewski J.K. (2012). Correlative nanomechanical profiling with super-resolution F-actin imaging reveals novel insights into mechanisms of cisplatin resistance in ovarian cancer cells. Nanomedicine.

[10] Cersosimo U., Sgorbissa A., Foti C., Drioli S., Angelica R., Tomasella A., Picco R., Semrau M.S., Storici P., Benedetti F. (2015). Synthesis, characterization, and optimization for in vivo delivery of a nonselective isopeptidase inhibitor as new antineoplastic agent. J. Med. Chem..

[11] Tomasella A., Blangy A., Brancolini C. (2014). A receptor-interacting protein 1 (RIP1)-independent necrotic death under the control of protein phosphatase PP2A that involves the reorganization of actin cytoskeleton and the action of cofilin-1. J. Biol. Chem..

[12] Barnes B.M., Nelson L., Tighe A., Burghel G.J., Lin I.H., Desai S., McGrail J.C., Morgan R.D., Taylor S.S. (2021). Distinct transcriptional programs stratify ovarian cancer cell lines into the five major histological subtypes. Genome Med..

[13] Burridge K.M., Parnell R.F., Kearns M.M., Page R.C., Konkolewicz D. (2021). Two-Distinct Polymer Ubiquitin Conjugates by Photochemical Grafting-From. Macromol. Chem. Phys..

[14] De Alwis Watuthanthrige N., Reeves J.A., Dolan M.T., Valloppilly S., Zanjani M.B., Ye Z., Konkolewicz D. (2020). Wavelength-Controlled Synthesis and Degradation of Thermoplastic Elastomers Based on Intrinsically Photoresponsive Phenyl Vinyl Ketone. Macromolecules.

[15] Jalal S., Shi S., Acharya V., Huang R.Y., Viasnoff V., Bershadsky A.D., Tee Y.H. (2019). Actin cytoskeleton self-organization in single epithelial cells and fibroblasts under isotropic confinement. J. Cell Sci..

[16] Raudenska M., Kratochvilova M., Vicar T., Gumulec J., Balvan J., Polanska H., Pribyl J., Masarik M. (2019). Cisplatin enhances cell stiffness and decreases invasiveness rate in prostate cancer cells by actin accumulation. Sci. Rep..

[17] Shen Y., Schmidt B.U.S., Kubitschke H., Morawetz E.W., Wolf B., Kas J.A., Losert W. (2020). Detecting heterogeneity in and between breast cancer cell lines. Cancer Converg..

[18] Xu W., Mezencev R., Kim B., Wang L., McDonald J., Sulchek T. (2012). Cell stiffness is a biomarker of the metastatic potential of ovarian cancer cells. PLoS ONE.

[19] Swaminathan V., Mythreye K., O’Brien E.T., Berchuck A., Blobe G.C., Superfine R. (2011). Mechanical stiffness grades metastatic potential in patient tumor cells and in cancer cell lines. Cancer Res..

[20] Nagayama M., Haga H., Kawabata K. (2001). Drastic change of local stiffness distribution correlating to cell migration in living fibroblasts. Cell Motil. Cytoskelet..

[21] Lautscham L.A., Kammerer C., Lange J.R., Kolb T., Mark C., Schilling A., Strissel P.L., Strick R., Gluth C., Rowat A.C. (2015). Migration in Confined 3D Environments Is Determined by a Combination of Adhesiveness, Nuclear Volume, Contractility, and Cell Stiffness. Biophys. J..

[22] Yoshiya N. (1986). Establishment of a cell line from human ovarian cancer (undifferentiated carcinoma of FIGO classification) and analysis of its cell-biological characteristics and sensitivity to anticancer drugs. Nihon Sanka Fujinka Gakkai Zasshi.

[23] Wilson A.P., Dent M., Pejovic T., Hubbold L., Radford H. (1996). Characterisation of seven human ovarian tumour cell lines. Br. J. Cancer.

[24] Louie K.G., Behrens B.C., Kinsella T.J., Hamilton T.C., Grotzinger K.R., McKoy W.M., Winker M.A., Ozols R.F. (1985). Radiation survival parameters of antineoplastic drug-sensitive and -resistant human ovarian cancer cell lines and their modification by buthionine sulfoximine. Cancer Res..

[25] Baguley B.C., Marshall E.S., Whittaker J.R., Dotchin M.C., Nixon J., McCrystal M.R., Finlay G.J., Matthews J.H., Holdaway K.M., van Zijl P. (1995). Resistance mechanisms determining the in vitro sensitivity to paclitaxel of tumour cells cultured from patients with ovarian cancer. Eur. J. Cancer.

[26] Morimoto H., Yonehara S., Bonavida B. (1993). Overcoming tumor necrosis factor and drug resistance of human tumor cell lines by combination treatment with anti-Fas antibody and drugs or toxins. Cancer Res..

[27] Buick R.N., Pullano R., Trent J.M. (1985). Comparative properties of five human ovarian adenocarcinoma cell lines. Cancer Res..

[28] Benard J., Da Silva J., De Blois M.C., Boyer P., Duvillard P., Chiric E., Riou G. (1985). Characterization of a human ovarian adenocarcinoma line, IGROV1, in tissue culture and in nude mice. Cancer Res..

[29] Ciotti S., Sgarra R., Sgorbissa A., Penzo C., Tomasella A., Casarsa F., Benedetti F., Berti F., Manfioletti G., Brancolini C. (2018). The binding landscape of a partially-selective isopeptidase inhibitor with potent pro-death activity, based on the bis(arylidene)cyclohexanone scaffold. Cell Death Dis..

[30] Du Y., Lin J., Zhang R., Yang W., Quan H., Zang L., Han Y., Li B., Sun H., Wu J. (2019). Ubiquitin specific peptidase 5 promotes ovarian cancer cell proliferation through deubiquitinating HDAC2. Aging.

[31] Azzalini E., Tierno D., Bartoletti M., Barbazza R., Giorda G., Puglisi F., Cecere S.C., Losito N.S., Russo D., Stanta G. (2022). AKT Isoforms Interplay in High-Grade Serous Ovarian Cancer Prognosis and Characterization. Cancers.

[32] Zonderland J., Wieringa P., Moroni L. (2019). A quantitative method to analyse F-actin distribution in cells. MethodsX.

[33] Sum C.S., Nickischer D., Lei M., Weston A., Zhang L., Schweizer L. (2014). Establishing a High-content Analysis Method for Tubulin Polymerization to Evaluate Both the Stabilizing and Destabilizing Activities of Compounds. Curr. Chem. Genom. Transl. Med..

[34] Rotsch C., Radmacher M. (2000). Drug-induced changes of cytoskeletal structure and mechanics in fibroblasts: An atomic force microscopy study. Biophys. J..

[35] Frixen U.H., Behrens J., Sachs M., Eberle G., Voss B., Warda A., Lochner D., Birchmeier W. (1991). E-cadherin-mediated cell-cell adhesion prevents invasiveness of human carcinoma cells. J. Cell Biol..

[36] Bai H., Li H., Li W., Gui T., Yang J., Cao D., Shen K. (2015). The PI3K/AKT/mTOR pathway is a potential predictor of distinct invasive and migratory capacities in human ovarian cancer cell lines. Oncotarget.

[37] Yi B.R., Kim T.H., Kim Y.S., Choi K.C. (2015). Alteration of epithelial-mesenchymal transition markers in human normal ovaries and neoplastic ovarian cancers. Int. J. Oncol..

[38] Luttman J.H., Colemon A., Mayro B., Pendergast A.M. (2021). Role of the ABL tyrosine kinases in the epithelial-mesenchymal transition and the metastatic cascade. Cell Commun. Signal..

[39] Rafehi S., Ramos Valdes Y., Bertrand M., McGee J., Prefontaine M., Sugimoto A., DiMattia G.E., Shepherd T.G. (2016). TGFbeta signaling regulates epithelial-mesenchymal plasticity in ovarian cancer ascites-derived spheroids. Endocr.-Relat. Cancer.

[40] Gil-Henn H., Patsialou A., Wang Y., Warren M.S., Condeelis J.S., Koleske A.J. (2013). Arg/Abl2 promotes invasion and attenuates proliferation of breast cancer in vivo. Oncogene.

[41] Wang H., Guo S., Kim S.J., Shao F., Ho J.W.K., Wong K.U., Miao Z., Hao D., Zhao M., Xu J. (2021). Cisplatin prevents breast cancer metastasis through blocking early EMT and retards cancer growth together with paclitaxel. Theranostics.

[42] Wang H., Tao L., Jin F., Gu H., Dai X., Ni T., Feng J., Ding Y., Xiao W., Qian Y. (2017). Cofilin 1 induces the epithelial-mesenchymal transition of gastric cancer cells by promoting cytoskeletal rearrangement. Oncotarget.

[43] Kasza K.E., Nakamura F., Hu S., Kollmannsberger P., Bonakdar N., Fabry B., Stossel T.P., Wang N., Weitz D.A. (2009). Filamin A is essential for active cell stiffening but not passive stiffening under external force. Biophys. J..

[44] Gladilin E., Ohse S., Boerries M., Busch H., Xu C., Schneider M., Meister M., Eils R. (2019). TGFbeta-induced cytoskeletal remodeling mediates elevation of cell stiffness and invasiveness in NSCLC. Sci. Rep..

[45] Kumari A., Shonibare Z., Monavarian M., Arend R.C., Lee N.Y., Inman G.J., Mythreye K. (2021). TGFbeta signaling networks in ovarian cancer progression and plasticity. Clin. Exp. Metastasis.

[46] Yu H., Lim K.P., Xiong S., Tan L.P., Shim W. (2013). Functional morphometric analysis in cellular behaviors: Shape and size matter. Adv. Healthc. Mater..

[47] Thomas G., Burnham N.A., Camesano T.A., Wen Q. (2013). Measuring the mechanical properties of living cells using atomic force microscopy. J. Vis. Exp..

[48] Bonin S., Pracella D., Barbazza R., Dotti I., Boffo S., Stanta G. (2019). PI3K/AKT Signaling in Breast Cancer Molecular Subtyping and Lymph Node Involvement. Dis. Markers.

