# Supplementary material for: Nanomechanical Characterization of Ovarian Cancer Cell Lines as a Marker of Response to 2c Treatment
Source: Int J Mol Sci. 2023 Apr 13;24(8):7230. doi: 10.3390/ijms24087230 (PMC10139025; doi:10.3390/ijms24087230)
Supplement: Supplementary file 1 [file ijms-24-07230-s001.zip › ijms-2317482-supplementary.pdf]

Cell Line	Average Young's Modulus Pop #1 (kPa)	Average Young's Modulus Pop #2 (kPa)
**SKOV3**	0.23 ± 0.17	0.92 ± 0.41
**HEY**	0.24 ± 0.20	1.04 ± 0.47
**OVCAR8**	0.3 ± 0.13	0.70 ± 0.22
**TYKNU CpR**	0.24 ± 0.12	0.60 ± 0.12
**OVCAR4**	0.41 ± 0.26	1.34 ± 0.48

Table S1: Average Young's Modulus (E) and standard deviation of the two populations in cell lines with bimodal distribution. Pop#1: lowest-stiffness population, Pop#2: highest stiffness population.

Cell Line (Sample size)	Morphological Classification (Circularity; Aspect Ratio)
**OAW42 (97)**	E (0.89 ± 0.03; 1.37 ± 0.16)
**IGROV1 (103)**	E (0.94 ± 0.01; 1.14 ± 0.10)
**SKOV3 (107)**	F (0.46 ± 0.13; 3.26 ± 0.63)
**TYKNU (100)**	F (0.43 ± 0.21; 3.44 ± 1.49)
**TYKNU CpR (102)**	F (0.44 ± 0.17; 3.11 ± 1.07)
**HEY (117)**	F (0.27 ± 0.03; 5.25 ± 1.51)
**OVCAR8 (103)**	E (0.74 ± 0.13; 1.91 ± 0.42)
**OVCAR4 (105)**	E (0.81 ± 0.15; 1.41 ± 0.20)

Table S2: Morphological classification of cell lines. For the morphological classification, the threshold values to discriminate between fibroblastic-like and epithelial-like morphology are 0.74 and 1.91 for circularity and aspect ratio respectively. E: Epithelial-like; F: Fibroblastic-like.

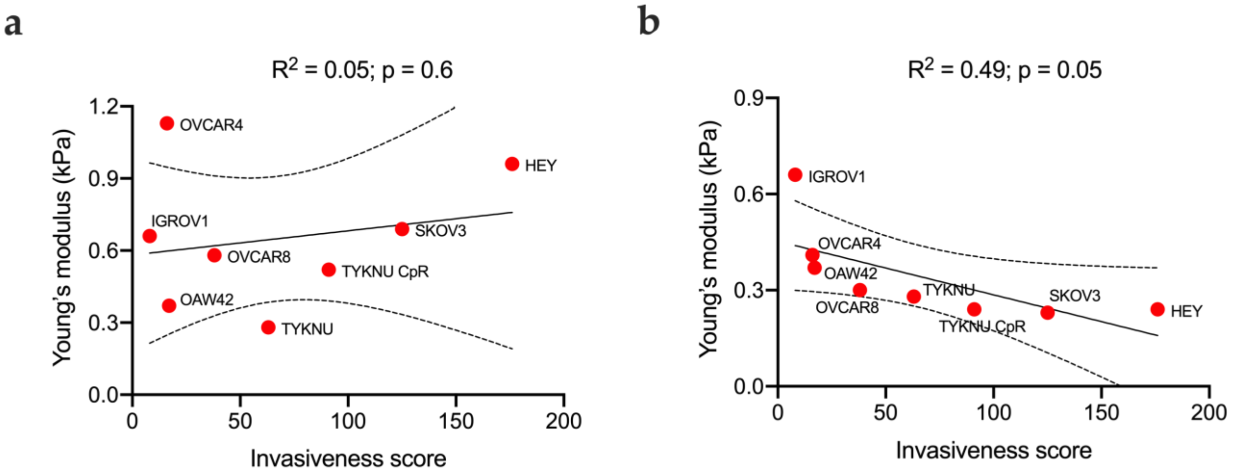

Figure S1: Scatterplot of the average number of invasive cells in function of the average Young's Modulus (**a**); Scatterplot of the average number of invasive cells in function of the average Young's Modulus, considering for cell lines with bimodal distribution only the lowest-stiffness population (**b**). Dotted lines represent the 95% confidence interval.

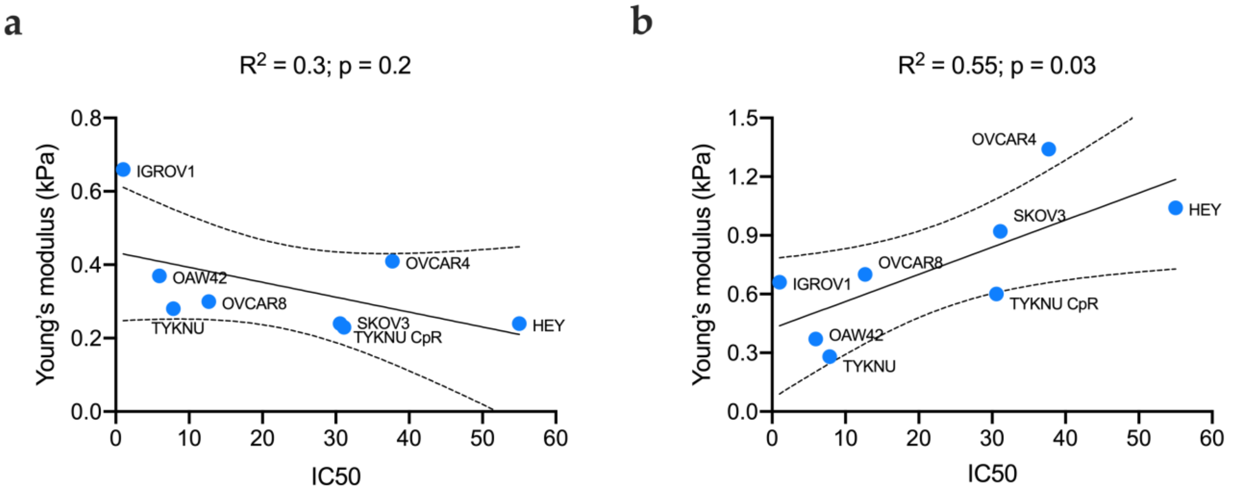

Figure S2: Scatterplot of the IC50 in function of the average Young's Modulus, considering for cell lines with bimodal distribution only the lowest-stiffness population (**a**); scatterplot of the IC50 in function of the average Young's Modulus, considering for cell lines with bimodal distribution only the highest-stiffness population (**b**). Dotted lines represent the 95% confidence interval.

Cell Line	E Pop #1 before 2c (kPa)	E Pop #1 after 2c (kPa)	CV% Pop #1	E Pop #2 before 2c (kPa)	E Pop #2 after 2c (kPa)	CV% Pop #2
**HEY**	0.24 ± 0.20	0.21 ± 0.19	9.43	1.04 ± 0.47	0.96 ± 0.26	5.66
**OVCAR4**	0.41 ± 0.26	0.19 ± 0.13	51.85	1.34 ± 0.48	0.98 ± 0.28	21.94
**TYKNU CpR**	0.24 ± 0.12	0.14 ± 0.10	37.22	0.60 ± 0.12	0.44 ± 0.17	21.76

Table S3: Effect of 2c on the Young modulus of the lowest (Pop #1) and highest (Pop #2) stiffness population in cell lines retaining a bimodal pattern after treatment. E Pop #1: average Young modulus of the "softer" population; E Pop #2: average Young modulus of the "stiffer" population; CV% = coefficient of variation.

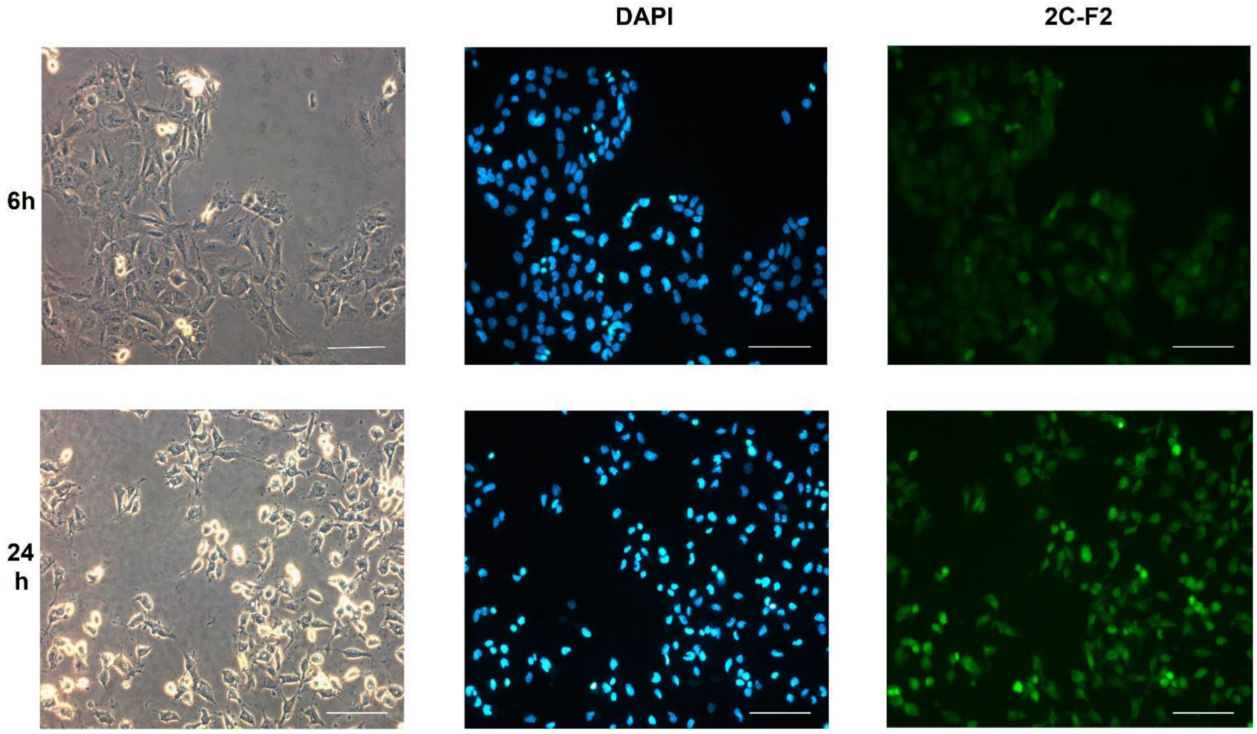

Figure S3: Fluorescent images of OVCAR4 after 2C-F2 treatment at 6 and 24 hours from treatment (3rd column). In figure are reported the raw images (1st column) and the DAPI-marked nuclei images (2nd column). All images were taken at a magnification of 20x. The scale bar corresponds to 100 μm.

MMP1	PPP2CA	AKT1	MMP8
TGFB1	PIK3CB	MMP3	WNT4
MTOR	SMARCA2	EEF1A2	MMP2
ABL2	MMP11	IGF1	PDGFA
HRAS	CCNE1	APC	SNAI1
AKT3	TGFBI	ITGB1	MAPK1
VIM	TUBG1	SMAD2	COL5A1
TWIST2	RHOT1	INF2	KRT8
ZEB1	MYD88	CTNNB1	E2F2
TWIST1	FN1	MMP14	AKT2
E2F1	ITGA5	MUC1	CCND3
CDH1	TUBB3	OCLN	TGFB2
SMARCAD1	TMSB4X	CDH2	TUBB1
PIK3CA	BRAF	EEF1A1	FLNA
ACTA2	CDC25C	FGFR1	COL5A2
CFL1	CLDN5	DSP	PTEN
NRAS	KRAS	KRT18	RB1
ZEB2	ACTB	IGF2	CDK2
MMP13	MKI67	NF1	KRT9
SNAI2	MMP9	VTN	ITGB2

Table S4: Panel of genes used for differential expression analysis. RNAseq data were extracted from CCLE Database.
